# Cryogenic auriculotherapy reduces pain and opioid use in patients undergoing endoscopic carpal tunnel release surgery: a retrospective analysis

**DOI:** 10.3389/fpain.2026.1722394

**Published:** 2026-02-24

**Authors:** Carine Chaix-Couturier, Christian Couturier, Ata Murat Kaynar, Bouchra Benkessou, Henri Weickmans, Stephanie Nuan, Jacques E. Chelly

**Affiliations:** 1 Clinic Bizet, Paris, France; 2 Clinic Arago Paris, Paris, France; 3 Department of Anesthesiology and Perioperative Medicine, University of Pittsburgh School of Medicine, Pittsburgh, PA, United States; 4 Institute Rafael, Center for Complementary Medicine, Levallois Perret, France

**Keywords:** auriculotherapy, carpal tunnel surgery, opioids, pain, perioperative pain management

## Abstract

**Background:**

Effective perioperative pain management for patients undergoing hand surgery is critical because of the risk of opioid misuse. Complementary approaches, including auriculotherapy (AT), for perioperative pain management have gained increasing attention. This study was designed to assess the analgesic properties of AT in patients undergoing endoscopic carpal tunnel surgery (CTR).

**Materials and methods:**

This retrospective study analyzed medical records of patients who underwent unilateral ambulatory CTR performed by a single surgeon under a regional block plus sedation, with or without the use of AT using a cryogenic approach. All procedures were performed between 1 October 2016 and 30 May 2017. A total of 38 consecutive patients were included in the analysis. At discharge, patients received a standard analgesic prescription for 3 days and were asked to record pain scores, using a verbal scale (0–10), and analgesic consumption on postoperative days (POD) 1, 2, and 3. The primary end points were overall consumption—expressed as the area under the curve (AUC) for oral morphine equivalent (OME) in mg—and pain scores between POD1 and POD3. Secondary end points included pain and opioid and non-opioid analgesic consumption. Data are presented as median (interquartile range) (25–75 percentile); *p* < 0.05 was considered significant.

**Results:**

A total of 38 consecutive patients were included in the analysis. There were 19 patients in the AT group (16 women and three men), with a mean age 54 ± 7.9 years, and 19 patients in the control group (15 women and four men), with a mean age of 57 ± 12.8 years. The use of cryo-AT was associated with a 44% reduction in OME consumption (0.0 [0.0; 10.0] OME mg in the AT group vs. 10 mg [0.0; 30.0] OME mg in the control group; *p* = 0.015) and lower pain scores (AUC POD1– 3; 0.33 [0.0;1.0] in the AT group vs. 1.67 [0.67; 2.3] in the control group; *p* = 0.0061). Naproxen consumption was not significantly different. At 21 days, no patient required further medication.

**Discussion:**

Our study suggests that cryogenic AT is an effective complementary alternative to opioids to control postoperative pain following CTR. Further investigations are required to confirm these findings.

## Background

Carpal tunnel release (CTR) surgery involves cutting the transverse carpal ligament to widen the carpal tunnel and reduce pressure on the median nerve, either via open surgery with an incision in the palm of the hand to access the transverse ligament or via endoscopic surgery with smaller incisions. Endoscopic surgery has been demonstrated to be less invasive than traditional open surgery, allowing the patient to experience less pain and a faster return to work (1, 2).

Although it has been established that the use of opioids is essential for optimal recovery (1, 2), due to the current opioid crisis and the increased risk of opioid use disorder (3) in surgical patients, the use of complementary approaches has been emphasized (4). Auriculotherapy (AT) is a form of acupuncture that focuses on the exclusive use of the ear as a way of treatment. AT is based on the unique innervation of the ear by cranial nerves, including the trigeminal nerve, the vagal nerve, the glossopharyngeal nerve, and the facial nerve (6). Each of these nerves innervates a specific area of the ear. Although evidence suggests that AT was used centuries ago, the technique was largely abandoned in the 19th century and reintroduced by Dr. Paul Nogier in the 1950s (5). Dr. Nogier's contributions included the concept that the ear contains a projection of the entire body—from the head down—and the presentation of one of the first human ear cartographies. This established the neurophysiological and neuropathological basis of AT. David Alimi (2002) established the neurophysiological bases of AT using functional magnetic resonance imaging (6, 7). His findings were later confirmed by Romoli et al. (8).

AT can be performed using different techniques, including needles (9), acupressure (10), laser (11), and more recently, a cryogenic approach (cryo) (12). Cryo-AT has demonstrated effectiveness in controlling cancer pain (13) and xerostomia (14). However, additional evidence is required to support the use of cryo-AT in reducing opioid use postoperative pain following orthopedic surgery.

This retrospective study was designed to assess the effectiveness of cryo-AT as an alternative to opioids following endoscopic CTR surgery. We hypothesized that cryo-AT performed prior to surgery would result in a significant decrease in opioid requirement and pain in patients undergoing endoscopic CTR surgery.

## Materials and methods

### Study design

This was a retrospective analysis of data collected between October 2016 and May 2017 from a single center (Bizet Clinic, Paris, France). The study was reviewed and approved by the Adene Group and the Institutional Review Board. The protocol was conducted in accordance with the Declaration of Helsinki and registered on the Clinicaltrials.gov website (NCT06693167; 18 November 2024). Cryo-AT was performed according to STRICTA guidelines (https://www.equator-network.org/reporting-guidelines/consort-stricta/) by an experienced physician trained at the *Diplome Inter-Universitaire d'Auriculotherapie-Neuromodulation Auriculaire* of Paris-Saclay University, France.

This retrospective study compared data obtained from the medical record of patients undergoing a single unilateral ambulatory endoscopic CTR performed by one surgeon (CC) under regional block plus sedation, with or without the use of auriculotherapy using a cryogenic approach (cryo-AT) for the management of postoperative pain.

Participants: Only patients who underwent an ambulatory isolated unilateral endoscopy CTR surgery with or without cryo-AT at one location (Clinic Bizet, Paris, France) were eligible. Consent for AT was incorporated into the surgical consent process after the patients received detailed information about the procedure.

Patients who underwent multiple surgeries and open CTR were excluded.

### Procedures

Anesthesia: Surgery was performed under axillary brachial plexus block using an ultrasound-guided approach and sedation.

**Figure 1:**
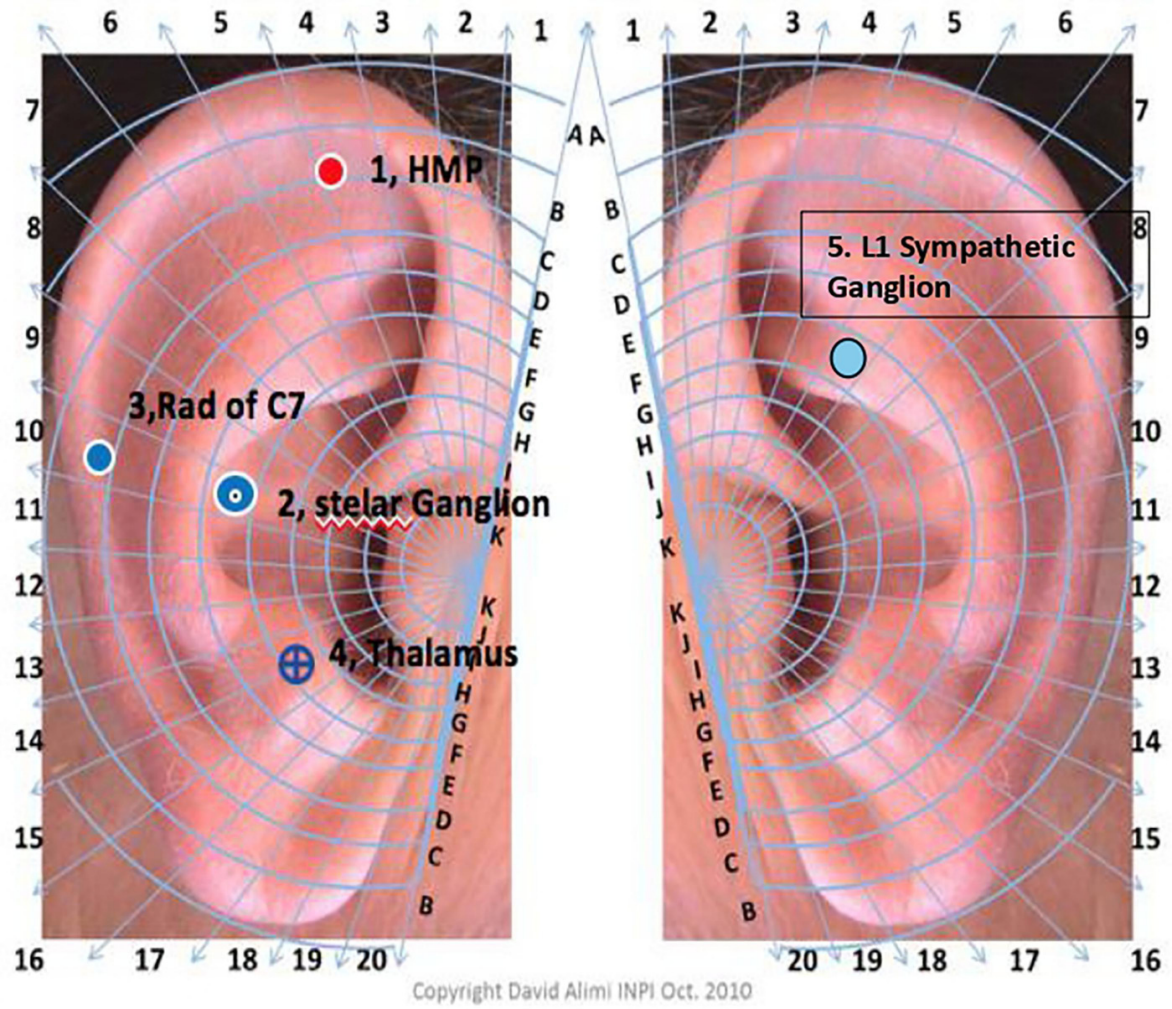
Auriculotherapy used for the auriculotherapy group. 1. Hand Master Point. 2. Stellate Ganglion. 3. Spinal cord of C7 = Rad of C7. 4. Thalamus. 5. L1 Sympathetic Ganglion.

**Figure 2:**
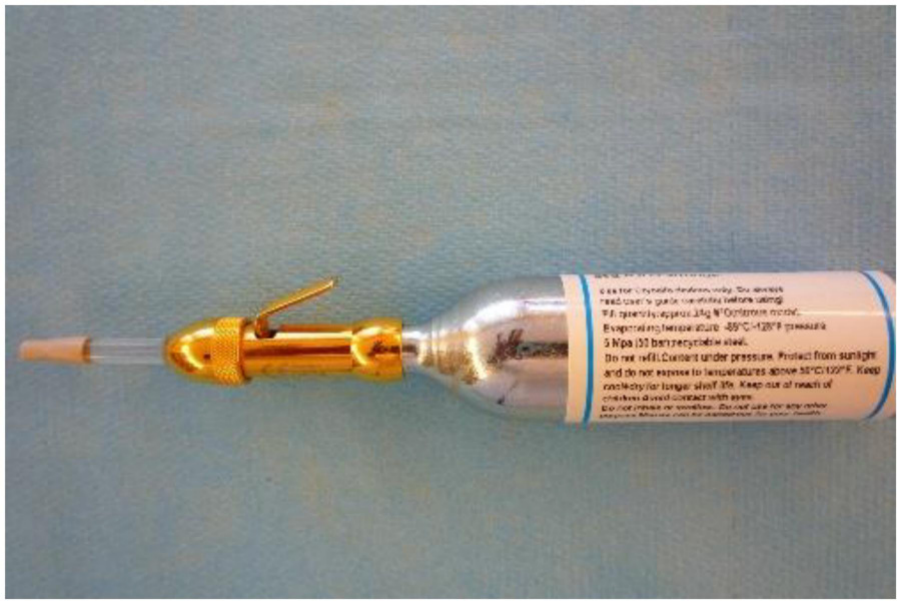
Cryopunctor comprised a canister of nitrous and an injector allowing to deliver a 2-s jet of gas at the level of each auricular point.

Cryo-AT treatment: Cryo-AT treatment included four ear points (Hand Master Point, Stellate Ganglion, C7 Radiculomere, and Thalamus) ipsilateral to the surgical site and one ear point (L1 Sympathetic ganglion) on the contralateral side to the surgical site (Figure 1). First, the ears were disinfected with alcohol. Each ear point was located using a digital microvoltmeter (Premio 20 DT Sedatelec, France) based on Dr. Alimi's cartography (15). Treatment consisted of a 2-s jet of nitrous oxide gas administered using a cryopunctor (Figure 2, Matauris, France). Cryo-AT was performed on the day of surgery, prior to patient transfer to the operating room.

Surgical procedure: CTR was performed by one surgeon (CC) using an endoscopic approach. The surgeon made one small opening in the wrist, and occasionally another at the level of the arm. These cuts were small, about a half inch each. Through these holes, a small camera was introduced to guide the surgeon to the location where the ligament was cut. Each patient was discharged within 2–3 h after surgery.

Standard postoperative prescription for pain: At discharge, at the surgeon’s request, each patient was prescribed 10 pills of acetaminophen 300 mg + opium 10 mg (oral morphine equivalent (OME in mg) + 30 mg caffeine and 10 pills of naproxen 550 mg. Each patient was also requested to wear an orthosis at night and during any painful activity during the day, and advised that they could return to their normal activities with regular use of their hand and wrist.

### Data collected

At the surgeon’s request, each patient was requested to record analgesic consumption and pain level using a verbal analog scale (0 = no pain to 10 = worst possible pain) on postoperative day (POD) 1 (24 h from discharge), 2, 3, and 21 (time of the follow-up visit). Data were documented in the medical record of each patient.

### Statistical analysis

The statistical analysis included descriptive statistics for the overall study population and for the cryo-AT and control groups.

#### Primary outcomes

Primary outcomes included overall analgesic consumption during the first 3 days following surgery, expressed as OME (mg), naproxen (mg), and pain scores using the area under the curve from postoperative day (POD) 1 to POD3 (AUC POD1–3).

#### Secondary outcomes

Secondary outcomes included opioid (OME, mg), naproxen consumption (OME, mg), and pain scores on POD1, POD2, and POD3, and analgesic consumption at POD21.

Data distribution was assessed using the Shapiro–Wilk test for normality. Data were analyzed using a Kruskal–Wallis *H* test when non-normally distributed (primary outcome) and a Mann–Whitney *U* test when normally distributed (secondary outcome). To account for multiple comparisons stemming from the analysis of secondary outcomes at three separate time points (POD1, POD2, and POD3), a Bonferroni correction was applied. Data are presented as median (interquartile range) due to the non-normal distribution (25–75 percentile). Alpha was set up at 0.05.

Statistical analyses were performed using GraphPad Prism (Version 10.6.1, GraphPad Software, Inc., La Jolla, CA, USA).

## Results

**Figure 3:**
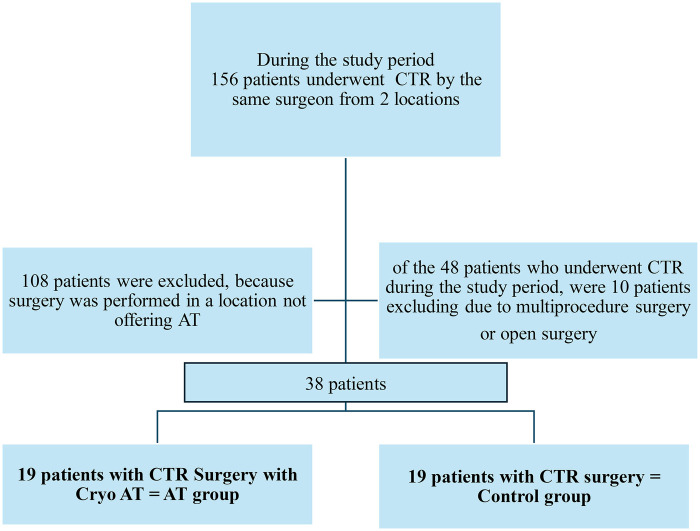
Study flow diagram.

During the reference period, a total of 156 patients underwent CTR by the same surgeon at two different locations. However, since cryo-AT was only offered at one location (Clinic de Bizet, Paris, France), the study population comprised the total number of patients who underwent CTR at that location (*n* = 48 patients). Ten patients were excluded from the analysis as they either underwent multiple procedure surgeries (*n* = 9) or open CTR (*n* = 1). Therefore, the population included in the analysis was based on an overall group of 38 patients, including 19 patients in the AT group and 19 in the control group. Figure 3 presents the study flow diagram.

No data were missing from the database. The patient cohort included 16 women and three men with a mean age of 54 ± 7.9 years in the AT group versus 15 women and four men with a mean age of 57 ± 12.8 years in the control group (Table 1).

**Table 1:** Characteristics of patients.

Groups of patients	Mean age	Female (%)	Baseline pain levels	Baseline analgesic consumption (mg)
Cryo-AT	54 years ±7.9	84	1.0 [0.0; 2.5]	10.0 [0.0; 24.0]
Control	57 years ±12.8	79	3.0 [1.0; 6.0]	19.6 [10.0; 30.0]

### Primary outcomes

The use of cryo-AT was associated with a 44% decrease in OME consumption (0.0 [0.0; 10.0] OME mg in the AT group vs. 10 mg [0.0; 30.0] OME mg in the control group; (*p* = 0.015)). The use of cryo-AT was associated with an 80% decrease in pain (AUC POD1–3; 0.33 [0.0;1.0] in the AT group vs. 1.67 [0.67; 2.3] in the control group; *p* = 0.0061).

**Figure 4:**
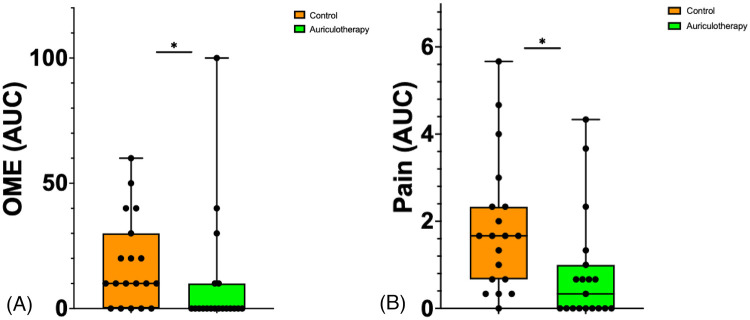
**(A)** Change in OME scores using the area under the curve (AUC) between POD1 and POD3. Data are presented as median [25%; 75%], filled circles presenting individual patient. *p = 0.015. **(B)** Change in pain scores using the area under the curve (AUC) between POD1 and POD3. Data are presented as median [25%; 75%], filled circles presenting individual patient, *p = 0.0061.

Figure 4A illustrates OME (mg) and Figure 4B illustrates pain expressed as AUC between POD1 and POD3. Supplementary Table 1 presents details of opioid consumption OME (mg) in the AT group and in the control group on POD1, POD2, and POD 3 following discharge from the hospital.

### Secondary outcomes

**Figure 5:**
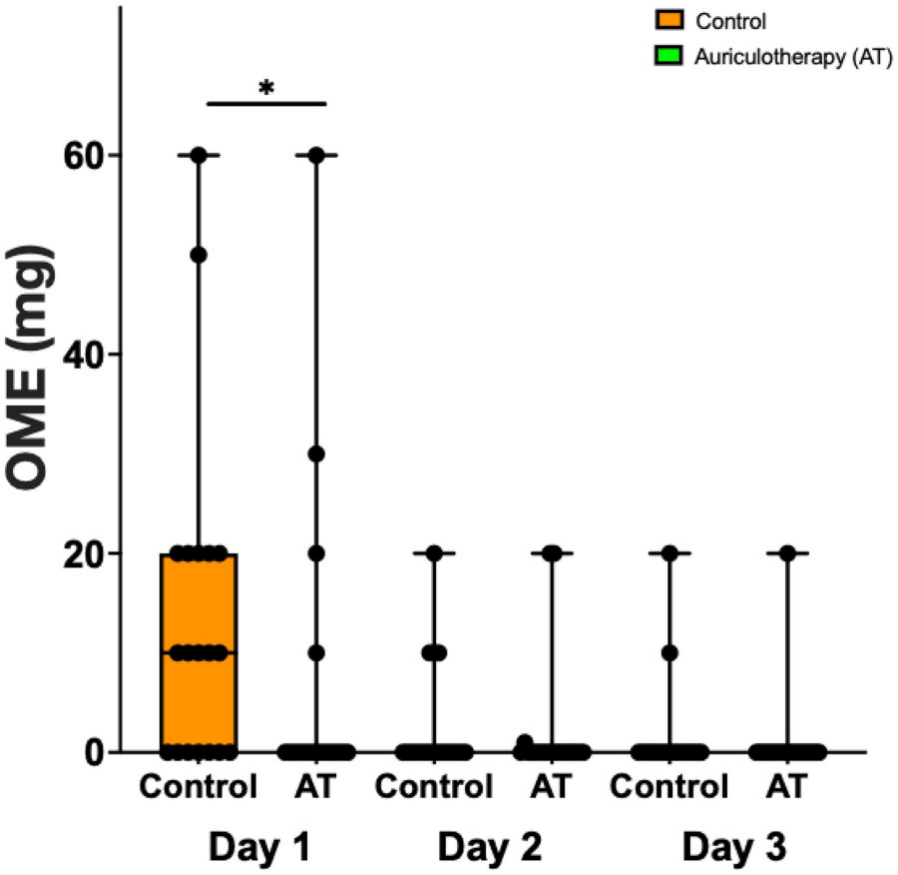
OME consumption (mg) in the AT and the control group on POD1, POD2, and POD3 following endoscopic CTR. Data are presented as median [25%; 75%], filled circles presenting individual patient. *p = 0.0263).

Opioid consumption in the AT group and in the control group on POD1, POD2, and POD3 following endoscopic CTR is represented in Figure 5. Reduction in opioid consumption for the AT group was significantly lower compared to the control group only on POD1 (0.0 [0.0; 0.0] vs. 10.0 [0.0; 20.0], *p* = 0.0263).

**Figure 6:**
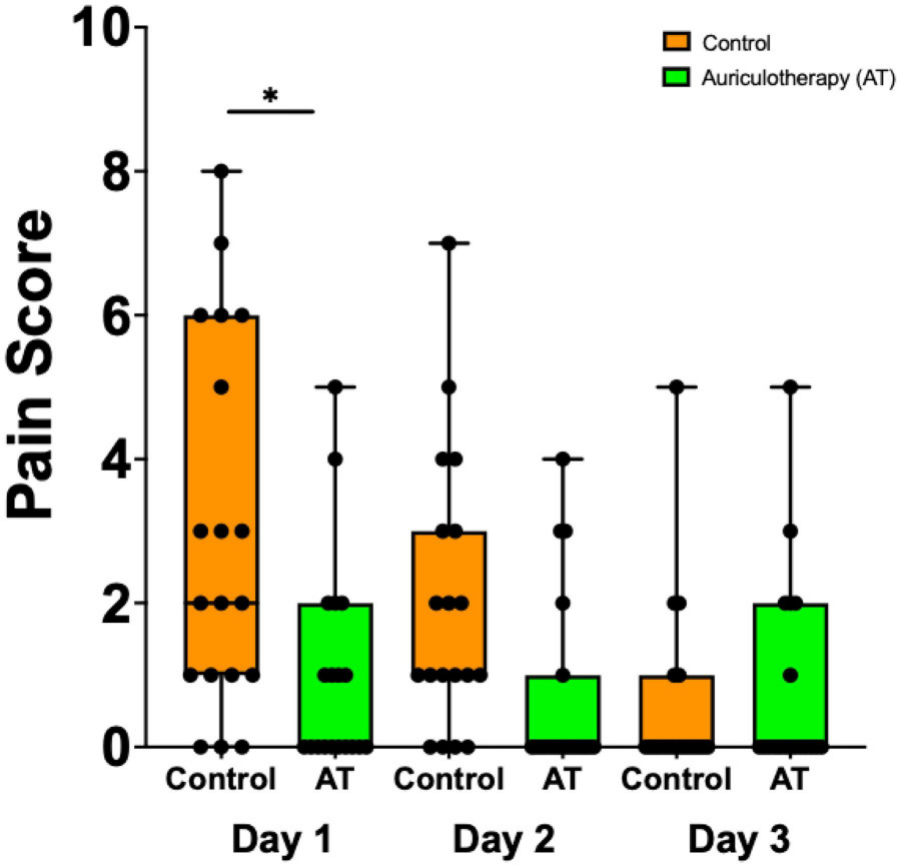
Pain scores in the AT group and the control group on POD1, POD2, and POD3 following endoscopic CTR. Data are presented as median [25%; 75%], filled circles presenting individual patient. *p = 0.0043.

Figure 6 present pain scores using (0 = no pain to 10 = worst possible pain) in the AT group and in the control group on POD1, POD2, and POD 3. The use of cryo-AT was associated with a significant reduction in pain on POD1. Supplementary Table 2 presents pain scores (0 = no pain and 10 = worst possible pain) of the AT group and the control group on POD1, POD2, and POD3 following the discharge from the hospital.

**Figure 7:**
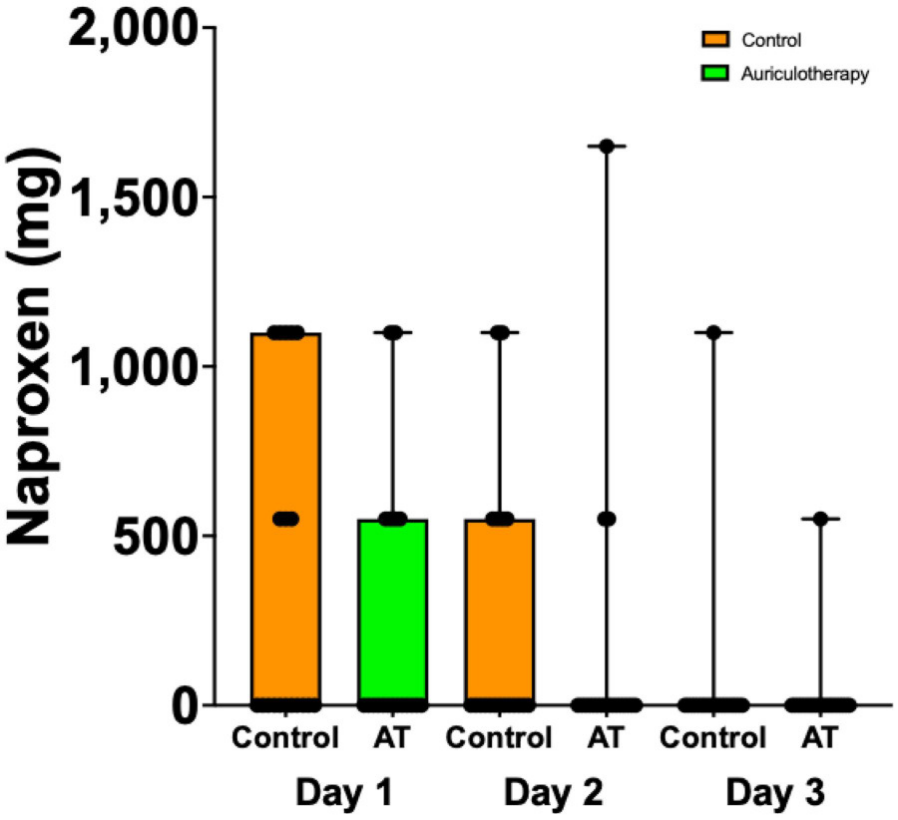
Naproxen consumption (mg) in AT group and control group on POD1, POD2, and POD3.

Naproxen consumption was not significantly different among the groups on POD1 (0.0 [0.0; 1,100.0] control group vs. 0.0 [0.0; 550.0] AT group), POD2 (0.0 [0.0; 550.0] control group vs. 0.0 [0.0; 0.0] AT group), and POD3 (0.0 [0.0; 0.0] control group vs. 0.0 [0.0; 0.0] AT group) (Figure 7). On POD21, no patient required any analgesic medication. Supplementary Table 3 Provides details of naproxen (mg) consumption (naproxen; mg) in the AT group and in the control group on POD1, POD2, and POD3 and overall consumption (Total) following discharge from the hospital.

## Discussion

Originating in China (16), auriculotherapy has been considered a part of acupuncture for centuries. However, the resurgence of auriculotherapy began with Dr. Paul Nogier in the 1940s, giving Europe an opportunity to play a more important role in the development and recognition of modern auriculotherapy. In June 1987, the World Health Organization acknowledged auriculotherapy as a specialty, and in 1990, the first international auriculo-nomenclature of ear points was proposed at an international meeting in Lyon. In 2010, the World Federation of Acupuncture Society discussed the topic of auriculotherapy extensively among Chinese and European experts, and in September 2011 in London, Dr. David Alimi proposed his cartography at the World Federation of Chinese Medicine Societies (15). Dr. Alimi's cartography was adopted by representatives from Europe, America, China, Russia, and Africa. Two main hypotheses have been proposed to explain the mechanisms of auriculotherapy: The first one is the Chinese/German hypothesis. It considers the ear as a continuation of the body’s 12 meridians—6 yang and 6 yin—with specific ear cartographies. The second hypothesis was proposed by David Alimi and is based on neurophysiology.

Auriculotherapy is based on the principle that ascendant and descendant spine pathways between the brain and the periphery exist, which constantly carry information about the peripheral homeostasis. These pathways include a proportion of neurofibers that transit in the ear, specifically at the level of each auricular point. This allows ear points to maintain equilibrium with the peripheral and the central nervous systems through the cranial nerves and the superficial plexus innervating the ear. Furthermore, each ear point exists in two configurations: a physiological configuration and a pathological configuration. The point in a pathologic configuration is representative of a peripheral pathologic condition. The treatment aims to eliminate the pathological configuration with the use of needles, laser, and cryogenic application. This generates a stimulation of the terminal nerve, innervating the point which is transmitted to the nucleus of the corresponding cranial nerve and subsequently to other brain structure. Stimulations are generated by the brain and transmitted to both a specific ear point and the corresponding part of the body. This results in the ear point being regenerated in its physiologic configuration and elimination of symptoms. For example, If the patient suffers of pain, the pain is reduced or even eliminated through the modulation of pain pathway.

Part of these findings were presented at the 9th International Auriculotherapy Symposium in Singapore on 10–12 August 2017. Our data indicated that the use of cryo-AT was associated with a significant reduction in opioid consumption and pain scores, suggesting that cryo-AT represents an effective alternative to opioids following endoscopic CTR surgery. Compared to traditional acupuncture needles, the cryogenic approach is less invasive. Additionally, this technique can be used in patients with a phobia of needles (including children).

More recently, similar benefits of cryo-AT have been reported in patients undergoing rotator cuff shoulder surgery (17).

Although pain levels reported in our study were similar to the pain levels reported by Ilyas et al. following endoscopic CTR release (18), their cohort demonstrated higher analgesic consumption than ours. This raises concerns over analgesic prescriptions to surgical patients (19). Non-opioid analgesics, including NSAIDs such as naproxen, are frequently prescribed for postoperative pain management after CTR surgery. Typically, NSAIDs often represent an alternative to opioids. However, they carry a serious side-effect profile, which includes myocardial ischemia, gastric bleeding, and renal insufficiency, requiring careful consideration when selecting postoperative pain regimens. In contrast, complementary techniques such as AT have demonstrated an established safety profile, making them interesting alternatives, even when compared to NSAIDs (20, 21)

The limitations of our study include a small sample size, the retrospective nature of our study, the involvement of only a single surgeon, and the fact that CTR represents a model of mild to moderate pain. However, the recorded data provide an interesting first level of evidence to assess the value of cryo-AT as an alternative to reduce postoperative pain and opioid use. These findings served as the foundation for the development of two randomized prospective studies to further evaluate the role that AT as an alternative to opioids and other analgesics in postoperative pain management.

## Conclusions

This preliminary retrospective study suggests that cryo-AT may be an effective complementary approach for perioperative pain management, extending beyond the duration of a nerve block and providing the opportunity to reduce postoperative opioid use. AT therefore represents a viable alternative for managing postoperative pain and reducing opioid use. However, further prospective randomized studies in a larger group of patients are required to confirm these initial findings.

## Data Availability

The datasets presented in this study can be found in online repositories. The names of the repository/repositories and accession number(s) can be found in the article/Supplementary Material.

## Supplementary material

Supplementary Table 1 Opioid consumption OME (mg) in the AT group and in the control group on POD1, POD2, and POD 3 following discharge from the hospital.

Supplementary Table 2 Pain scores using (0=no pain and 10=worst possible pain) in the AT group and in the control group on POD1, POD2, and POD 3 following the discharge from the hospital.

Supplementary Table 3 Naproxen (mg) consumption (naproxen; mg) in the AT group and in the control group on POD1, POD2, and POD3 and overall consumption (total) following discharge from the hospital.
