# Correction: Cryogenic-auriculotherapy reduces pain and opioid use in patients undergoing endoscopic carpal tunnel release surgery; a retrospective analysis

**DOI:** 10.3389/fpain.2026.1824847

**Published:** 2026-03-27

**Authors:** Carine Chaix-Couturier, Christian Couturier, Ata Murat Kaynar, Bouchra Benkessou, Henri Weickmans, Stephanie Nuan, Jacques E. Chelly

**Affiliations:** 1 Clinic Bizet, Paris, France; 2 Clinic Arago Paris, Paris, France; 3 Department of Anesthesiology and Perioperative Medicine, University of Pittsburgh School of Medicine, Pittsburgh, PA, United States; 4 Institute Rafael, Center for Complementary Medicine, Levallois Perret, France

**Keywords:** auriculotherapy, carpal tunnel surgery, opioids, pain, perioperative pain management

Author name misspelled:

Author Ata Murat Kaynar was erroneously spelled as Murat A. Kaynar.

The original version of this article has been updated.

