# Supplementary material for: Cryogenic auriculotherapy reduces pain and opioid use in patients undergoing endoscopic carpal tunnel release surgery: a retrospective analysis
Source: Front Pain Res (Lausanne). 2026 Feb 24;7:1722394. doi: 10.3389/fpain.2026.1722394 (PMC12971898; doi:10.3389/fpain.2026.1722394)
Supplement: Supplementary file 1 [file Datasheet1.pdf]

Supplemental material

Supplemental Table 1. Opioid consumption OME(mg) in the AT group) and in the control group on POD1, POD2 and POD 3 following discharge from the hospital

	POD1		POD 2		POD 3		Total	
Group	AT Group	Control group	AT Group	Control group	AT Group	Control group	AT Group	Control group
Patient 1	0	0	0	0	0	0	0	0
2	0	10	0	0	0	0	0	10
3	0	20	1	10	0	0	10	30
4	60	0	20	0	20	0	100	0
5	0	20	0	0	0	0	0	20
6	30	10	0	0	0	0	30	10
7	0	0	0	10	0	10	0	20
8	0	10	0	0	0	0	0	10
9	0	0	0	0	0	0	0	0
10	0	0	0	0	0	0	0	0
11	20	0	20	0	0	0	40	0
12	10	20	0	0	0	20	10	40
13	0	60	0	0	0	0	0	60
14	0	10	0	0	0	0	0	10
15	0	0	0	10	0	0	0	10
16	0	20	0	0	0	0	0	20
17	0	10	0	0	0	0	0	10
18	0	50	0	0	0	0	0	50
19	0	20	0	20	0	0	0	40

Supplemental Table 2 Pain scores using (0=no pain and 10= worst possible pain) in the AT group and in the control group on POD1, POD2 and POD 3 following the discharge from the hospital

	**POD 1**		**POD 2**		**POD 3**		**Average**	
GROUPS	AT group	Control group	AT group	Control Group	AT group	Control group	AT group	Control group
Patient 1	1	8	1	1	0	0	1	3
2	0	3	0	1	2	0	1	1
3	1	6	0	7	1	1	1	5
4	5	0	3	1	5	0	4	0
5	4	3	4	3	3	0	4	2
6	0	1	0	0	0	0	0	0
7	0	0	0	2	0	0	0	1
8	1	1	0	0	0	0	0	0
9	0	2	0	2	0	1	0	2
10	1	1	0	1	2	0	1	1
11	2	0	2	0	0	0	1	0
12	2	3	0	2	0	2	1	2
13	0	7	0	5	0	5	0	6
14	2	2	3	3	2	0	2	2
15	0	1	0	4	0	0	0	2
16	0	5	0	0	0	0	0	2
17	0	2	0	1	0	0	0	1
18	0	6	0	1	0	0	0	2
19	0	6	0	4	0	2	0	4

Supplemental Table 3. Naproxen (mg) consumption (naproxen; mg) in the AT group and in the control group on POD 1, POD 2 and POD 3 and Overall consumption (Total) following discharge from the hospital

	POD 1		POD 2		POD 3		Total	
Group	AT Group	Control group	AT Group	Control group	AT Group	Control group	AT Group	Control group
Patient1	0	550	0	0	0	0	0	550
2	0	0	0	0	0	0	0	0
3	550	2	0	550	0	0	550	1,650
4	550	0	550	0	550	0	1,650	0
5	0	1,100	0	0	0	0	0	1,100
6	550	0	0	0	0	0	550	0
7	0	0	0	1,100	0	1,100	0	2,200
8	550	550	0	0	0	0	550	550
9	0	0	0	0	0	0	0	0
10	1,100	0	550	0	0	0	1,650	0
11	1,100	0	1,650	0	0	0	2,750	0
12	0	0	0	0	0	0	0	0
13	0	550	0	0	0	0	0	550
14	0	0	0	0	0	0	0	0
15	0	0	0	550	0	0	0	550
16	0	0	0	550	0	0	0	550
17	0	1,100	0	550	0	0	0	1,650
18	0	1,100	0	0	0	0	0	1,100
19	0	1,100	0	1,100	0	0	0	2,200
